# Global intracranial arterial tortuosity is associated with intracranial atherosclerotic burden

**DOI:** 10.1038/s41598-024-61527-z

**Published:** 2024-05-17

**Authors:** Mi-Yeon Eun, Ha‑Na Song, Jong‑Un Choi, Hwan‑Ho Cho, Hyung Jun Kim, Jong-Won Chung, Tae-Jin Song, Jin-Man Jung, Oh‑Young Bang, Gyeong‑Moon Kim, Hyunjin Park, David S. Liebeskind, Woo-Keun Seo

**Affiliations:** 1 Department of Neurology, School of Medicine, Kyungpook National University, Daegu, South Korea; 2 Department of Neurology, Graduate School, Korea University, Seoul, South Korea; 3 Department of Neurology and Stroke Center, Samsung Medical Center, Sungkyunkwan University School of Medicine, 81, Irwon-Ro, Gangnam-Gu, Seoul, 06351 South Korea; 4 Department of Digital Health, SAIHST, Sungkyunkwan University School of Medicine, Seoul, South Korea; 5 Department of Electronics Engineering, Incheon National University, Incheon, South Korea; 6 Department of Neurology, Seoul Hospital, Ewha Womans University College of Medicine, Seoul, South Korea; 7 Department of Neurology, Korea University Ansan Hospital, Korea University College of Medicine, Ansan, South Korea; 8 Department of Electrical and Computer Engineering, Sungkyunkwan University, Suwon, South Korea; 9 Center for Neuroscience Imaging Research, Institute for Basic Science (IBS), Suwon, South Korea; 10 Department of Neurology, University of California in Los Angeles, Los Angeles, CA USA

**Keywords:** Tortuosity, Intracranial atherosclerosis, Stroke, Neurology, Atherosclerosis

## Abstract

The effect of arterial tortuosity on intracranial atherosclerosis (ICAS) is not well understood. This study aimed to evaluate the effect of global intracranial arterial tortuosity on intracranial atherosclerotic burden in patients with ischemic stroke. We included patients with acute ischemic stroke who underwent magnetic resonance angiography (MRA) and classified them into three groups according to the ICAS burden. Global tortuosity index (GTI) was defined as the standardized mean curvature of the entire intracranial arteries, measured by in-house vessel analysis software. Of the 516 patients included, 274 patients had no ICAS, 140 patients had a low ICAS burden, and 102 patients had a high ICAS burden. GTI increased with higher ICAS burden. After adjustment for age, sex, vascular risk factors, and standardized mean arterial area, GTI was independently associated with ICAS burden (adjusted odds ratio [adjusted OR] 1.33; 95% confidence interval [CI] 1.09–1.62). The degree of association increased when the arterial tortuosity was analyzed limited to the basal arteries (adjusted OR 1.48; 95% CI 1.22–1.81). We demonstrated that GTI is associated with ICAS burden in patients with ischemic stroke, suggesting a role for global arterial tortuosity in ICAS.

## Introduction

Arterial tortuosity is a common angiographic finding in a variety of vascular systems, including the cerebral arteries^1^. It has been suggested that arterial tortuosity is correlated with aging, female sex, hypertension, and genetic disorders^2^. Previous studies have reported that a reduced axial stress or arterial wall degradation results in mechanical instability and arterial wall remodeling, leading to vascular elongation and tortuosity^1–3^. Arterial tortuosity can induce blood flow alterations and shear stress on the arterial wall^1^ and may be reciprocally associated with atherosclerosis^4,5^.

Intracranial atherosclerosis (ICAS) is one of the major causes of ischemic stroke, particularly in Asians^6^. Although common atherosclerotic risk factors such as hypertension, diabetes mellitus, and dyslipidemia increase ICAS risk, there are some differences between ICAS and general atherosclerosis in terms of its risk factors, with dyslipidemia being less important and metabolic syndrome increasing the risk^7^. Furthermore, intracranial arteries have greater interindividual variation in trajectory^8^ and may be more prone to tortuosity due to their anatomical features^2^. However, the effect of arterial tortuosity on ICAS has not been well established.

There are several methods for measuring arterial tortuosity^9–12^; however, previous studies have typically measured the tortuosity of a single vessel of interest and evaluated its association with atherosclerosis. However, given that tortuosity is prominent at the arterial borders and that atherosclerosis is also prevalent at major arterial bifurcations or branching points, measuring of the tortuosity of a single vessel may not be adequate to assess the burden of atherosclerosis. We hypothesized that the tortuosity of the entire intracranial arteries may reflect ICAS burden. Therefore, we aimed to measure the global intracranial arterial tortuosity, assessed by an in-house vessel analysis software, and to evaluate the effect of global tortuosity index (GTI) of the entire intracranial arteries on ICAS burden in patients with acute ischemic stroke.

## Methods

### Study population

This research was supported by grants from the national research foundation of Korea (NRF), funded by the Korean government (MSIT). We performed a retrospective analysis on patient data collected from a prospective hospital-based stroke registry during the research project, covering the period from January 2017 to December 2019. The registry includes data on consecutive patients with acute ischemic stroke within 7 days of onset and includes demographic data, medical history, laboratory findings, stroke severity and etiology, and clinical outcomes. Demographic data and vascular risk factors were collected from the registry data and medical records.

This study included the following patients: (1) patients aged ≥ 20 years, (2) with a diagnosis of acute ischemic stroke confirmed by brain imaging, (3) who underwent magnetic resonance angiography (MRA). Exclusion criteria were as follows: (1) those with MRA that could not be analyzed and geometric features could not be extracted, (2) patients with other vascular pathologies other than atherosclerosis, such as dissection, central nervous system vasculitis, Moyamoya disease, or reversible cerebral vasoconstriction syndrome, (3) patients with arterial occlusion because the vascular pathology could not be identified.

The samsung medical center institutional review board approved this study (SMC-2021-04-137). Informed consent was waived due to the retrospective study design and anonymized data analysis by samsung medical center. This study was conducted ethically in accordance with the declaration of the world medical association declaration of Helsinki.

### Assessment of the burden of ICAS

Intracranial three-dimensional time-of-flight (TOF) MRA was performed using a 3.0 T magnetic resonance imaging system (philips medical systems) with a 32-element phased array receiver head coil. Scan parameters were as follows: TE, 4.59 ms; TR, 22 ms; flip angle, 23; FOV, 250 × 250 × 1.2 mm^3^; voxel size, 0.284 × 0.284 × 1.2 mm^3^; total acquisition time, 352 s; RBW, 130 Hz/pixel; GRAPPA factor, 3; 32 reference lines.

Two neurologists blinded to geometric features independently reviewed each patient’s intracranial TOF MRA image, and each intracranial artery was classified as having no significant atherosclerotic stenosis, significant atherosclerotic stenosis, or occlusion. Stenoses ≥ 50% were defined as significant stenosis. The following intracranial arteries were included in the imaging review: bilateral intracranial internal carotid artery (ICA), middle cerebral artery (MCA) branches M1 and M2, anterior cerebral artery (ACA) branches A1 and A2, posterior cerebral artery (PCA) branches P1 and P2, basilar artery (BA), and vertebral artery (VA). Cerebellar arteries (CbllA) were excluded from ICAS burden assessment due to frequent poor visualization or hypoplasia. Disagreements were resolved by consensus. After exclusion of cases with occlusion, the number of ICAS was calculated. The ICAS burden was stratified into no ICAS, low ICAS burden with one significant intracranial artery stenosis, and high ICAS burden with two or more intracranial artery stenoses.

### Assessment of global intracranial arterial tortuosity

Analysis of vessel geometric features was performed using an in-house vessel analysis software, the methods of which have been reported previously^13^. Detailed methods are presented in the Supplementary Methods. Arteries included in the analyses of geometric features were ICA, MCA, ACA, PCA, BA, VA, and cerebellar arteries, which include the superior cerebellar artery (SCA), anterior inferior cerebellar artery (AICA), and posterior inferior cerebellar artery (PICA).

Curvature is a geometric feature that quantifies the degree of bending in a line. For a line, it is calculated as the inverse of the radius of the osculating circle at a specific point $P(x,y,z)$. For a parametrically defined 3D curve, curvature $\kappa$ is computed using the following formula:

$$\kappa = \frac{{\sqrt {\left( {z^{\prime\prime}y^{\prime} - y^{\prime\prime}z^{\prime}} \right)^{2} + \left( {x^{\prime\prime}z^{\prime} - z^{\prime\prime}x^{\prime}} \right)^{2} + \left( {y^{\prime\prime}x^{\prime} - x^{\prime\prime}y^{\prime}} \right)^{2} } }}{{\left( {x^{^{\prime}2} + y^{^{\prime}2} + z^{^{\prime}2} } \right)^{3/2} }},$$

The apostrophes within the formula indicate derivatives. The first and second derivatives of x, y, and z for the centerline points can be computed through calculation of the gradient of the coordinates at adjacent points. The graphical representation of the curvature is shown in Supplementary Fig. 1.

Curvature was measured at all points and the mean curvature (curvature_m_) was calculated by averaging the curvatures in each arterial segment and in the whole intracranial arteries. The tortuosity index (TI) represents the standardized curvature_m_ for each artery, and the standardized curvature_m_ for all intracranial arteries is defined as the GTI. A positive standardized GTI value indicates higher overall arterial tortuosity compared to the study population average, while a negative value suggests lower tortuosity. Values approaching zero indicate tortuosity levels close to the population mean.

We measured the cross-sectional area at the centerline of each artery using our in-house vessel analysis software. In addition, the mean area (area_m_) was calculated by averaging the cross-sectional areas across all points within the entire intracranial arterial system.

Vessels that comprised the Circle of Willis were categorized as basal cerebral arteries and the others as distal arteries. To assess the global tortuosity of relatively large arteries on ICAS burden, we additionally defined GTIb as GTI analysis restricted to basal arteries only.

### Statistical analysis

Continuous variables were presented as mean ± standard deviation or median and interquartile range, as appropriate. Frequencies and percentages were calculated for the categorical variables. For continuous variables with less than 5% missing values, such as fasting blood glucose and total cholesterol, the mean values were imputed. For vascular geometric features, data were standardized to reduce the scale differences between features. Baseline characteristics and vessel geometric characteristics were compared between the three groups according to ICAS burden using chi-squared tests for categorical variables and analysis of variance or Kruskal–Wallis test for continuous variables.

Univariable and multivariable ordinal logistic regression analyses were performed to estimate the adjusted common odds ratio (OR) with 95% confidence interval (CI) of GTI for a shift towards more ICAS burden. Model 1 was adjusted for age and sex, and model 2 was adjusted by age, sex, and independent variables with a P-value < 0.1 in the univariable analysis. We also included the standardized area_m_ value in model 3, in addition to the variables in model 2. To assess for the existence of multi-collinearity, a variance inflation factor test was performed. Brant’s test was used to test for the assumption of parallel regression. We analyzed the effect of GTIb on ICAS burden in the same way. Subgroup comparisons of GTI on ICAS burden were performed and the interaction effect between GTI and patient demographics and vascular risk factors was assessed. Two-sided P-values of < 0.05 were considered statistically significant. Data analyses were performed using statistical software R version 4.3.0^14^ or SPSS for Windows, version 25.0 (IBM Corporation, Armonk, NY, USA).

## Results

### Baseline characteristics

**Figure 1:**
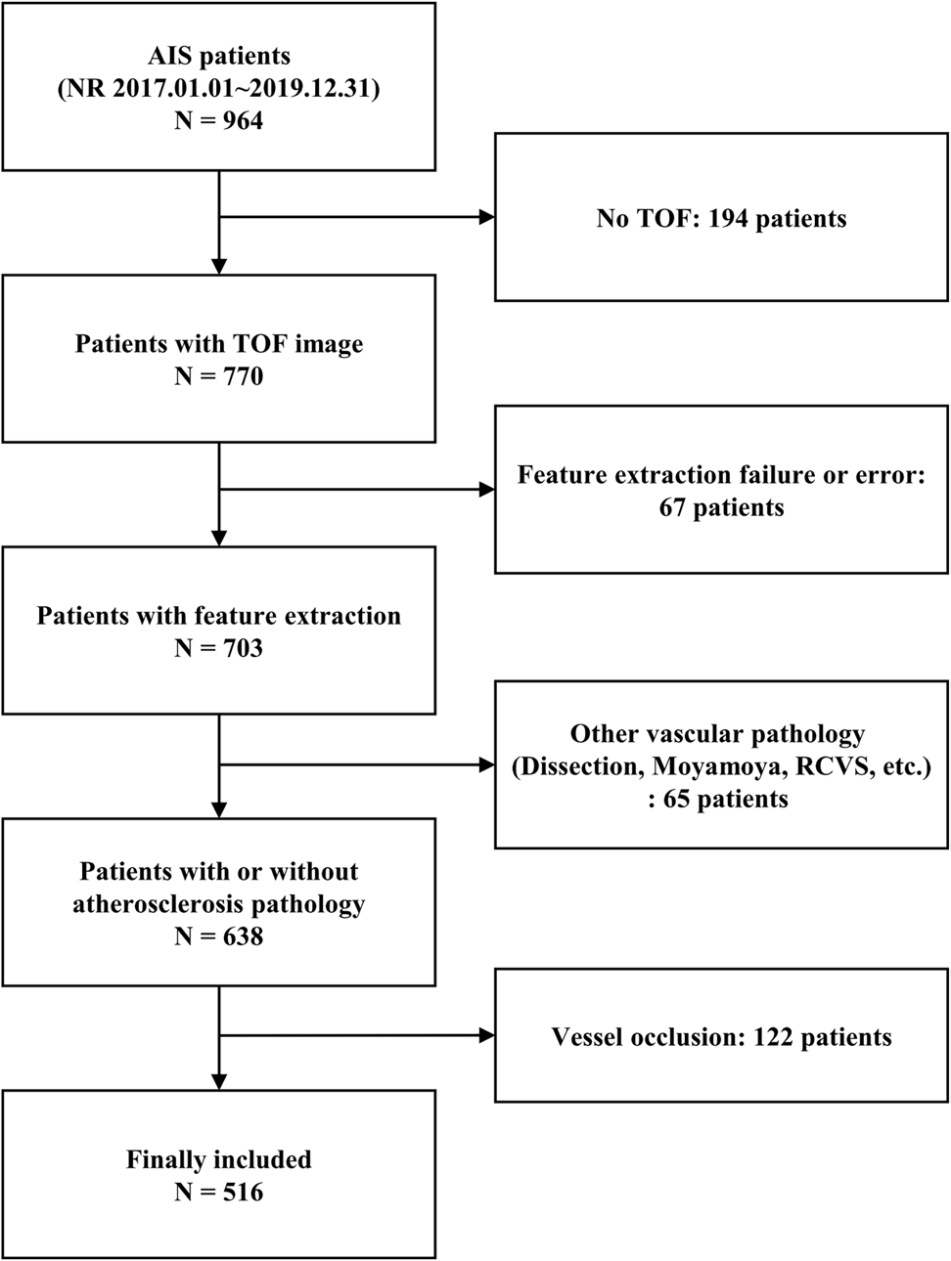
Selection of the study population.

A total of 964 patients with acute ischaemic stroke were screened between January 2017 and December 2019, of whom 770 patients underwent TOF MRA imaging. Sixty-seven patients were excluded because arterial feature extraction was unavailable due to poor image quality. We also excluded 65 patients with vascular anomalies other than atherosclerosis, such as dissection, Moyamoya disease, and reversible cerebral vasoconstriction syndrome, and 122 patients with major intracranial arterial occlusion (Fig. 1). Of the 516 participants (mean age 67.6 ± 13.0 years, 34.9% women) included, 274 patients had no ICAS, 140 patients had one ICAS (low ICAS burden), and 102 patients had two or more ICAS (high ICAS burden). The high ICAS burden group was older and had more comorbidities, such as hypertension, diabetes mellitus, and coronary artery disease. Details of the participants are summarized in Table 1.

**Table 1:** Baseline characteristics of the participants according to ICAS burden.

	No ICAS (N = 274)	Low ICAS (N = 140)	High ICAS (N = 102)	P-value
Age	66.0 ± 13.4	68.1 ± 12.0	71.1 ± 12.7	0.002
Male	184 (67.2)	86 (61.4)	68 (66.7)	0.492
BMI	23.9 ± 3.1	24.1 ± 3.6	24.7 ± 3.0	0.127
Height	165.0 ± 9.2	163.7 ± 8.6	161.9 ± 9.2	0.012
SBP	151.6 ± 28.1	156.7 ± 27.9	154.0 ± 27.8	0.209
DBP	87.2 ± 17.3	85.3 ± 17.2	81.7 ± 15.5	0.02
Hypertension	178 (65.0)	110 (78.6)	85 (83.3)	< 0.001
Diabetes mellitus	65 (23.7)	56 (40.0)	44 (43.1)	< 0.001
Dyslipidemia	159 (58.0)	92 (65.7)	68 (66.7)	0.167
Current smoking	63 (23.0)	33 (23.6)	16 (15.7)	0.256
Atrial fibrillation	57 (20.8)	17 (12.1)	15 (14.7)	0.066
Congestive heart failure	8 (2.9)	2 (1.4)	3 (2.9)	0.7
Peripheral arterial disease	1 (0.4)	1 (0.7)	4 (3.9)	0.028
Previous stroke or TIA	42 (15.3)	34 (24.3)	32 (31.4)	0.002
Coronary artery disease	21 (7.7)	16 (11.4)	22 (21.6)	0.001
Aortic arch complex atheroma	1 (0.4)	1 (0.7)	5 (4.9)	0.01
Hemoglobin	13.8 ± 2.0	13.6 ± 2.0	13.38 ± 2.1	0.259
WBC	7.61 ± 3.51	7.89 ± 2.58	7.38 ± 2.39	0.423
Platelet	222.9 ± 75.4	229.3 ± 97.8	219.5 ± 68.9	0.618
Fasting blood glucose	120.4 ± 43.1	121.1 ± 45.0	128.5 ± 50.1	0.283
Total cholesterol	173.4 ± 42.9	169.5 ± 44.7	167.6 ± 45.5	0.451
Triglyceride	154.64 ± 97.7	155.75 ± 88.0	160.11 ± 99.7	0.884
HDL-C	49.9 ± 14.5	47.2 ± 16.2	46.7 ± 13.6	0.082
LDL-C	112.0 ± 40.0	111.9 ± 39.7	1009.0 ± 40.9	0.793
hs-CRP	0.77 ± 3.05	0.55 ± 1.24	0.67 ± 1.56	0.673
TOAST				< 0.001
LAA	42 (15.3)	41 (29.3)	46 (45.1)	
CE	71 (25.9)	24 (17.1)	12 (11.8)	
SAO	96 (35.0)	40 (28.6)	23 (22.5)	
ODE	14 (5.1)	2 (1.4)	3 (2.9)	
UDE	51 (18.6)	33 (23.6)	18 (17.6)	
Prior antiplatelet agents	81 (29.6)	64 (45.7)	48 (47.1)	< 0.001
Prior statin use	77 (28.1)	53 (37.9)	45 (44.1)	0.007
Premorbid mRS	0.0 [0.0–0.0]	0.0 [0.0–0.0]	0.0 [0.0–0.0]	0.016
Initial NIHSS	2.0 [1.0–4.0]	3.0 [1.0–4.5]	3.0 [1.0–5.0]	0.027

Values are expressed as number (%), mean ± standard deviation, or median [interquartile range].

*ICAS* intracranial atherosclerosis, *SD* standard deviation, *BMI* body mass index, *SBP* systolic blood pressure, *DBP* diastolic blood pressure, *TIA* transient ischemic attack, *WBC* white blood cell, *HDL-C* high-density lipoprotein cholesterol, *LDL-C* low-density lipoprotein cholesterol, *hs-CRP* high-sensitivity c-reactive protein, *TOAST* trial of org 10,172 in acute stroke treatment classification, *LAA* large artery atherosclerosis, *CE* cardioembolism, *SAO* small artery occlusion, *ODE* other determined etiology, *UDE* undetermined etiology, *mRS* modified Rankin Scale, *NIHSS* national institutes of health stroke scale.

### Measurement of intracranial arterial tortuosity in study population

**Figure 2:**
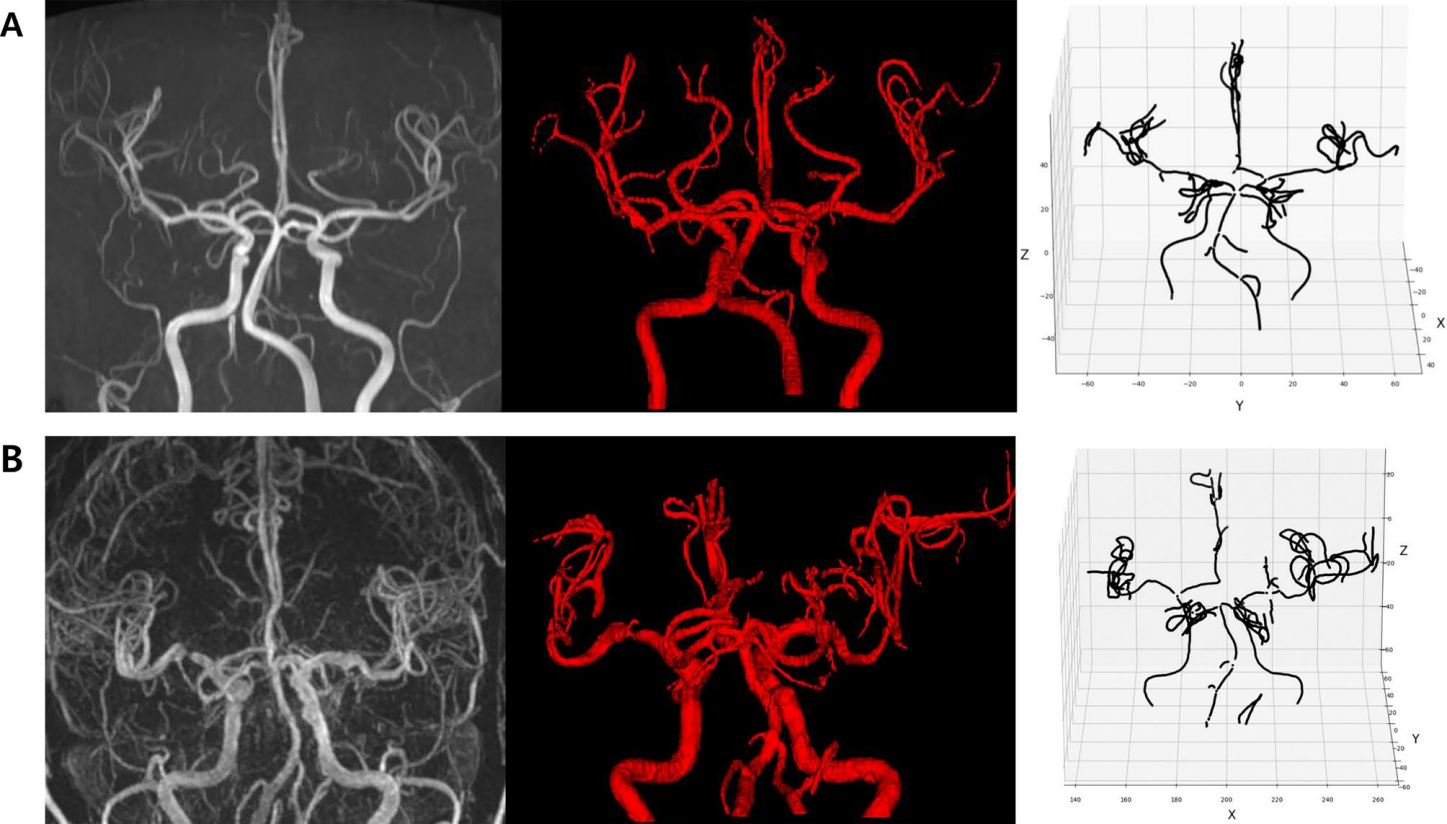
Analysis of intracranial arteries using an in-house vessel analysis software. (**A**) Intracranial time-of-flight (TOF) magnetic resonance angiography (MRA) image (FOV, 250 × 250 × 1.2 mm3; voxel size, 0.284 × 0.284 × 1.2 mm^3^) of a patient with normal intracranial arteries and image processed by region-growing and centerline extraction. (**B**) TOF MRA and processed image in patients with atherosclerosis of the right middle cerebral artery. *FOV* field of view.

Among 8772 screened intracranial arteries, 590 arteries exhibited significant stenosis (86 ICAs, 153 MCAs, 40 ACAs, 61 PCAs, 23 BAs, and 69 VAs). The in-house vessel analysis software extracted arterial features relatively well, and representative cases are demonstrated in Fig. 2. For each vessel, the ICA, basal and distal MCA had missing curvature values ranging from 0.2 to 3.9%. The basal and distal ACA had missing values ranging from 2.5 to 10.9%. In the posterior circulation, 18.4% of the left VA and 34.7% of the right VA were missing due to hypoplasia and dominance, and 18.4% of the BA was missing. For the PCA, missing values ranged from 2.3 to 2.5% for the basal PCA and from 37.6 to 39.3% for the distal PCA. The cerebellar artery was missing between 3.1 and 5.2%. The curvature_m_ of the intracranial arteries were mostly symmetric in the right and left intracranial arteries and the curvature_m_ of the BA was lowest. The intracranial arteries in the posterior circulation and the distal arteries tended to have relatively higher tortuosity (Supplementary Table 1).

### GTI in study population

**Figure 3:**
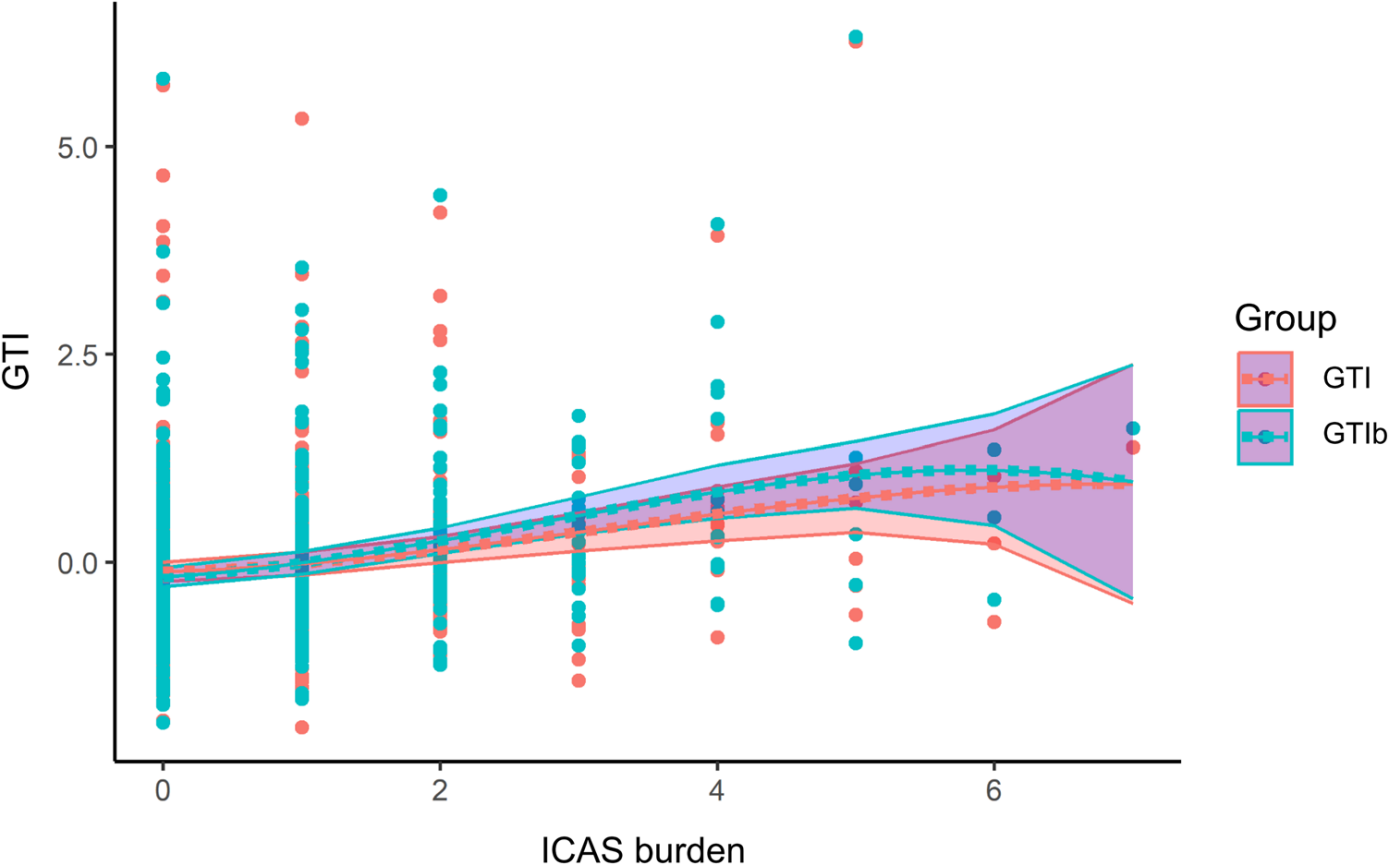
Distribution of global tortuosity index (GTI) and basal artery GTI (GTIb) values according to the number of stenoses in the intracranial arteries, plotted as a restricted cubic spline curve. The x-axis represents the number of stenoses in the intracranial arteries, while the y-axis displays the GTI values. Colored areas represent 95% confidence intervals.

GTI was higher in patients with large-artery atherosclerotic stroke than in those with non-large artery atherosclerotic stroke (0.13 ± 1.09 vs. − 0.04 ± 0.96), although this difference was not statistically significant (P-value = 0.08). Similarly, GTI was slightly higher in patients with coronary artery disease (0.18 ± 1.18 vs. − 0.02 ± 0.97; P-value = 0.153) and peripheral artery disease (0.53 ± 0.0.84 vs. − 0.01 ± 1.0; P-value = 0.195), but these differences were also not statistically significant. GTI increased in a dose-dependent manner with the number of ICAS (Fig. 3). GTI was significantly higher in the high ICAS group than in the low ICAS or no ICAS group (no ICAS, − 0.12 ± 0.93; low ICAS, − 0.02 ± 0.97; high ICAS, 0.33 ± 1.16; P < 0.001). This trend was also confirmed in GTIb, with the difference in tortuosity index between groups being greater in GTIb (no ICAS, − 0.18 ± 0.91; low ICAS, − 0.04 ± 0.93; high ICAS, 0.52 ± 1.15). Looking at the curvature_m_ of each vessel, TI of both ICAs, right basal MCA, BA, right basal PCA, and right CbllA were higher in the high ICAS group than in the no ICAS and low ICAS groups, similar to the GTI (Table 2). Standardized area_m_ was slightly higher in the high ICAS group than in the no ICAS and low ICAS groups (no ICAS, − 0.04 ± 0.89; low ICAS, 0.01 ± 0.85; high ICAS, 0.08 ± 1.40; P = 0.613). However, this was not statistically significant.

**Table 2:** Global tortuosity index (GTI) and tortuosity index (TI) of individual intracranial arteries based on ICAS burden.

	No ICAS (N = 274)	Low ICAS (N = 140)	High ICAS (N = 102)	P-value
GTI	− 0.12 ± 0.93	− 0.02 ± 0.97	0.33 ± 1.16	< 0.001
GTIb	− 0.18 ± 0.91	− 0.04 ± 0.93	0.52 ± 1.15	< 0.001
R ICA TI	− 0.13 ± 0.95	0.08 ± 0.99	0.24 ± 1.08	0.004
L ICA TI	− 0.13 ± 0.94	− 0.01 ± 0.87	0.36 ± 1.23	< 0.001
R bMCA TI	− 0.18 ± 0.75	0.05 ± 1.22	0.43 ± 1.12	< 0.001
L bMCA TI	− 0.08 ± 0.31	0.12 ± 1.84	0.04 ± 0.28	0.153
R bACA TI	− 0.06 ± 0.88	0.06 ± 1.22	0.09 ± 0.95	0.342
L bACA TI	0.01 ± 1.21	− 0.02 ± 0.72	0.00 ± 0.65	0.968
R dMCA TI	0.03 ± 1.35	− 0.06 ± 0.19	0.00 ± 0.27	0.683
L dMCA TI	− 0.07 ± 0.89	0.06 ± 1.02	0.10 ± 1.24	0.219
R dACA TI	− 0.05 ± 0.88	− 0.03 ± 0.65	0.17 ± 1.56	0.176
L dACA TI	− 0.02 ± 1.01	0.01 ± 0.91	0.05 ± 1.11	0.823
BA TI	− 0.09 ± 0.44	− 0.08 ± 0.40	0.38 ± 2.10	0.001
R VA TI	0.09 ± 1.25	− 0.14 ± 0.41	− 0.07 ± 0.66	0.164
L VA TI	− 0.07 ± 0.38	0.05 ± 1.22	0.14 ± 1.70	0.224
R bPCA TI	− 0.11 ± 1.08	− 0.02 ± 0.76	0.34 ± 0.99	0.001
L bPCA TI	− 0.06 ± 0.80	0.08 ± 1.33	0.05 ± 0.96	0.36
R dPCA TI	− 0.04 ± 0.89	− 0.03 ± 1.12	0.14 ± 1.11	0.454
L dPCA TI	0.00 ± 0.46	− 0.13 ± 0.38	0.21 ± 2.22	0.147
R CbllA TI	− 0.11 ± 0.93	− 0.01 ± 0.91	0.33 ± 1.22	0.001
L CbllA TI	− 0.06 ± 0.34	− 0.03 ± 0.39	0.21 ± 2.14	0.071

Values are mean ± standard deviation.

*ICAS* intracranial atherosclerosis, *GTI* global tortuosity index, *GTIb* basal artery GTI, *IQR* interquartile range, *ICA* internal carotid artery, *bMCA* basal middle cerebral artery, *dMCA* distal middle cerebral artery, *bACA* basal anterior cerebral artery, *dACA* distal anterior cerebral artery, *BA* basilar artery, *VA* vertebral artery, *bPCA* basal posterior cerebral artery, *dPCA* distal posterior cerebral artery, *CbllA* cerebellar artery.

### Effects of GTI on ICAS burden

Univariable analysis revealed that age, diastolic blood pressure, hypertension, diabetes mellitus, less atrial fibrillation, peripheral arterial disease, previous stroke or TIA, coronary artery disease, aortic arch complex atheroma, low high-density lipoprotein cholesterol, etiology of large artery atherosclerosis, previous antiplatelet agents use, previous statin use, initial NIHSS, and GTI were associated with greater ICAS burden (Supplementary Table 2).

To assess the effect of GTI on ICAS burden, we performed multivariable ordinal logistic regression analysis. In model 1, GTI was associated with greater ICAS burden (adjusted OR, 1.41; 95% CI 1.18–1.67). After adjustment for potential confounders, GTI remained as an independent predictor for greater ICAS burden in model 2 (adjusted OR, 1.36; 95% CI 1.12–1.65) and model 3 (adjusted OR 1.33; 95% CI 1.09–1.62) (Table 3). In addition to GTI, hypertension (adjusted OR 1.66; 95% CI 1.05–2.65), diabetes mellitus (adjusted OR, 1.71; 95% CI 1.14–2.55), previous stroke or TIA (adjusted OR 1.79; 95% CI 1.11–2.88), aortic arch complex atheroma (adjusted OR 7.01; 95% CI 1.14–42.92), large-artery atherosclerotic etiology relative to small-vessel occlusion etiology (adjusted OR 2.4; 95% CI 1.48–3.88), and initial national institutes of health stroke scale (adjusted OR 1.07; 95% CI 1.01–1.12) were significantly correlated with ICAS burden (Supplementary Table 2). Comparison of the effect on ICAS burden in different subgroups revealed a consistent trend with the overall effect. There was no significant interaction between GTI and subgroups (Supplementary Fig. 2).

**Table 3:** Effects of global tortuosity index (GTI) and GTIb on intracranial atherosclerotic burden.

	Adjusted OR (95% CI) (model 1)*	Adjusted OR (95% CI) (model 2)^†^	Adjusted OR (95% CI) (model 3)^‡^
GTI	1.41 (1.18–1.67)***	1.36 (1.12–1.65)**	1.33 (1.09–1.62)**
GTIb	1.66 (1.38–1.99)***	1.49 (1.22–1.81)***	1.48 (1.22–1.81)***

*ICAS* intracranial atherosclerosis, *OR* odds ratio, *CI* confidence interval, *GTI* global tortuosity index, *GTIb* basal artery GTI.

*Adjusted for age and sex.

***P < 0.001; **P < 0.01.

^†^Adjusted for age, sex, variable with P-value < 0.1 in the univariate analysis.

^‡^Adjusted for variables in model 2 and area_m_.

Univariable and multivariable analyses were also performed for the effect of GTIb on ICAS burden and the degree of association with ICAS burden was stronger for the GTIb (adjusted OR 1.48; 95% CI 1.22–1.81) than for the GTI (Table 3).

## Discussion

In this study, the tortuosity of intracranial arteries was measured according to the curvature extracted using in-house vessel analysis software. GTI was increased with higher ICAS burden and was independently associated with increased ICAS burden after adjustment for potential confounders and standardized area_m_. The effect of GTI on ICAS burden was consistent across subgroups. Notably, the effect of tortuosity on ICAS burden was stronger when the arteries for analysis were restricted to the basal cerebral arteries.

While systemic risk factors are crucial for atherosclerosis development, there has been a growing interest in hemodynamic and geometric factors^15,16^. Wall shear stress is a frictional stress applied in a direction parallel to the vessel wall. Disturbed shear stress is sensed by the endothelial mechanoreceptors and may contribute to atherosclerosis development by inducing inflammation and increasing susceptibility to vascular injury^15^. Atherosclerosis is more likely to occur in areas with low wall shear stress, while high wall shear stress may contribute to vulnerable plaque formation and plaque rupture. Arterial tortuosity can induce blood flow derangements such as flow shift, flow recirculation, and low oscillatory wall shear stress^17^. Thus, arterial tortuosity may be a geometric risk for atherosclerosis, which may explain the predilection for atherosclerotic lesions at arterial bifurcation sites.

This study demonstrated that GTI is increased and independently associated with ICAS burden, and this finding is consistent with previous studies reporting that arterial geometric risk is associated with atherosclerosis. In the carotid arteries, more atherosclerosis was noted characterized by kinking, tortuosity, and coiling^4^. Tortuosity has also been reported to be associated with atherosclerosis in the coronary, femoral, and intracranial arteries^11,12,18,19^.

Our study is relevant because geometric risk may be a more important issue in ICAS. The effect of lipids on the development of atherosclerosis in intracranial arteries is relatively small compared with extracranial or coronary arteries^7^. Conversely, intracranial arteries may have a relatively high degree of tortuosity^2,20^ because they are less anchored to the external structure, and have a small diameter, and usually have multiple branches^21^. Furthermore, the intracranial arteries have a relatively minimal outward remodeling and develop plaque protrusion into the lumen from an early stage^22^. This induces altered shear stress around the plaque and may reciprocally influence the tortuosity of intracranial arteries. Thus, the effect of tortuosity may be greater and more exaggerated in the intracranial arteries than in other vessels.

We developed a global index of tortuosity by tracking whole MRA-identifiable intracranial arteries and found that this had a significant impact on ICAS burden. Several studies have elucidated that arterial tortuosity is associated with atherosclerosis, dissection, and aneurysm development^11,23–25^. To determine disease associations, most studies measured TI in single relevant arteries. However, many atherosclerotic plaques can develop at bifurcations and at the arterial borders, and tortuosity tends to be greater at these locations. Therefore, this approach may be inappropriate because of the paucity of information regarding the tortuosity of the edge of intracranial arteries. Furthermore, tortuosity in one vessel may induce hemodynamic changes in distal arteries beyond that vessel^26,27^. It is hence difficult to correlate tortuosity measured in a specific vessel with ICAS burden. GTI in this study may overcome these shortcomings to provide a comprehensive assessment of the effect of tortuosity on the total ICAS burden.

Although tortuosity was calculated based on the centerline of the artery, hemodynamic changes due to tortuosity may be influenced by several factors including arterial cross-sectional area, shear stress or blood pressure. Therefore, we included area_m_ in our multivariable analyses to determine the effect of GTI on ICAS burden. The results indicated that area_m_ did not have a significant effect on ICAS burden, but it is necessary to consider a range of variables as factors affecting ICAS burden.

Another potential confounding factor was height. Taller individuals typically have longer intracranial arteries and this may influence the hemodynamic response to arterial tortuosity on ICAS. Our results demonstrated that lower height tended to be associated with ICAS burden, however, this was not statistically significant in the multivariable analysis.

In this study, the effect of tortuosity on ICAS burden is greater when GTI is restricted to the basal arteries. These results varied from our expectation that vessels with high tortuosity might have a greater effect on ICAS burden, as most distal arteries have larger mean curvature values compared to basal arteries. However, considering that atherosclerosis mainly occurs in large and medium-sized arteries, this seems plausible.

There are numerous methods for measuring vessel tortuosity^2,10–12^. Previous studies have usually assessed the arterial tortuosity using indirect methods^11,28^. However, these methods can be inaccurate and may not reflect the hemodynamic and geometric changes. In addition, previous methods require time and effort for manual measurement, rendering them difficult to use in clinical practice. We could automatically assess the tortuosity from the absolute mean curvature of the intracranial arteries on the 3D angiographic map using an in-house vessel analysis software. The results are consistent with visual inspection, with a higher tortuosity in the distal arteries and the lowest tortuosity in the basilar artery. Intracranial ICA tortuosity was relatively lower than in the previous study, probably due to the influence of the post-petrous straight segment of the ICA. A recent study reported that mean absolute curvature using computer modelling correlates best with coronary wall shear stress than other methods of assessing tortuosity^29^. Therefore, GTIs have the potential to elucidate the pathophysiology of ICAS and may be used to predict the progression of ICAS or to assess the effectiveness of treatment. Furthermore, the method used in our study provides information from each small segment of the artery. By combining and analyzing this information by vessel and anatomical structure, it is expected that more insight into vascular pathology can be gained.

Our study has several limitations. First, this study is a retrospective case-control study and may have potential residual confounding effects. Second, we only analyzed the effect of tortuosity on ICAS in patients with stroke; therefore, the results cannot be generalized to individuals without stroke. Third, some images could not be automatically assessed for tortuosity using in-house software due to poor image quality. However, these limitations may also be present in visual assessment and may be overcome through technological advances in image correction and improvement in software accuracy. Fourth, there is a discrepancy between the arteries assessed for ICAS burden and those included in the GTI calculations. ICAS burden was evaluated through manual review of MRA images for arteries such as ICA, MCA, ACA, PCA, BA, and VA, whereas GTI calculations included CbllA such as SCA, AICA, and PICA, which were often inadequately visualized on MRA. This discrepancy may be a limitation of our study, but also implies the potential of automated analysis software to overcome the challenges of manual visual assessment. Finally, our data lack the detailed information on whether ICAS was symptomatic or not, and whether the large artery atherosclerotic stroke according to the TOAST classification was categorized as extracranial or intracranial large artery atherosclerosis. Understanding the symptomatic status or the location of atherosclerosis may provide additional implication, which warrants further study. Despite these limitations, our study demonstrated the association between global intracranial arterial tortuosity and ICAS burden in patients with ischemic stroke, suggesting a role for geometric risk in ICAS.

## Supplementary Information

Supplementary Information.

## Data Availability

The study data are available from the corresponding author upon reasonable request and with the permission of all contributing authors.

## References

[1] Han, H. C. Twisted blood vessels: Symptoms, etiology and biomechanical mechanisms. *J. Vasc. Res.* **49**, 185–197. 10.1159/000335123 (2012). 22433458 10.1159/000335123 PMC3369246

[2] Ciurica, S. *et al.* Arterial tortuosity. *Hypertension* **73**, 951–960. 10.1161/HYPERTENSIONAHA.118.11647 (2019). 30852920 10.1161/HYPERTENSIONAHA.118.11647

[3] Jackson, Z. S., Dajnowiec, D., Gotlieb, A. I. & Langille, B. L. Partial off-loading of longitudinal tension induces arterial tortuosity. *Arterioscler. Thromb. Vasc. Biol.* **25**, 957–962. 10.1161/01.ATV.0000161277.46464.11 (2005). 15746437 10.1161/01.ATV.0000161277.46464.11

[4] Del Corso, L. *et al.* Tortuosity, kinking, and coiling of the carotid artery: Expression of atherosclerosis or aging?. *Angiology* **49**, 361–371. 10.1177/000331979804900505 (1998). 9591528 10.1177/000331979804900505

[5] Thomas, J. B. *et al.* Variation in the carotid bifurcation geometry of young versus older adults: Implications for geometric risk of atherosclerosis. *Stroke* **36**, 2450–2456. 10.1161/01.STR.0000185679.62634.0a (2005). 16224089 10.1161/01.STR.0000185679.62634.0a

[6] Wang, Y. *et al.* Prevalence and outcomes of symptomatic intracranial large artery stenoses and occlusions in China: The Chinese intracranial atherosclerosis (CICAS) study. *Stroke* **45**, 663–669. 10.1161/STROKEAHA.113.003508 (2014). 24481975 10.1161/STROKEAHA.113.003508

[7] Kim, J. S. & Bonovich, D. Research on intracranial atherosclerosis from the East and West: Why are the results different?. *J. Stroke* **16**, 105–113. 10.5853/jos.2014.16.3.105 (2014). 25328869 10.5853/jos.2014.16.3.105 PMC4200588

[8] Klostranec, J. M. & Krings, T. Cerebral neurovascular embryology, anatomic variations, and congenital brain arteriovenous lesions. *J. Neurointerv. Surg.* **14**, 910–919. 10.1136/neurintsurg-2021-018607 (2022). 35169032 10.1136/neurintsurg-2021-018607

[9] Metz, H., Murray-Leslie, R. M., Bannister, R. G., Bull, J. W. & Marshall, J. Kinking of the internal carotid artery. *Lancet* **1**, 424–426. 10.1016/s0140-6736(61)90004-6 (1961). 13769898 10.1016/s0140-6736(61)90004-6

[10] Bullitt, E., Gerig, G., Pizer, S. M., Lin, W. & Aylward, S. R. Measuring tortuosity of the intracerebral vasculature from MRA images. *IEEE Trans. Med. Imaging* **22**, 1163–1171. 10.1109/TMI.2003.816964 (2003). 12956271 10.1109/TMI.2003.816964 PMC2430603

[11] Kim, B. J. *et al.* Vascular tortuosity may be related to intracranial artery atherosclerosis. *Int. J. Stroke* **10**, 1081–1086. 10.1111/ijs.12525 (2015). 26061533 10.1111/ijs.12525

[12] Li, X. *et al.* Tortuosity of the superficial femoral artery and its influence on blood flow patterns and risk of atherosclerosis. *Biomech. Model. Mechanobiol.* **18**, 883–896. 10.1007/s10237-019-01118-4 (2019). 30652210 10.1007/s10237-019-01118-4

[13] Hong, S. W. *et al.* Automated in-depth cerebral arterial labelling using cerebrovascular vasculature reframing and deep neural networks. *Sci. Rep.* **13**, 3255. 10.1038/s41598-023-30234-6 (2023). 36828857 10.1038/s41598-023-30234-6 PMC9957982

[14] R Core Team. R: *A Language and Environment for Statistical Computing*. R Foundation for Statistical Computing, 2022) https://www.R-project.org/

[15] Kwak, B. R. *et al.* Biomechanical factors in atherosclerosis: Mechanisms and clinical implications. *Eur. Heart J.* **35**(3013–3020), 3020a–3020d. 10.1093/eurheartj/ehu353 (2014). 10.1093/eurheartj/ehu353 PMC4810806 25230814

[16] Morbiducci, U. *et al.* Atherosclerosis at arterial bifurcations: Evidence for the role of haemodynamics and geometry. *Thromb. Haemost.* **115**, 484–492. 10.1160/th15-07-0597 (2016). 26740210 10.1160/TH15-07-0597

[17] Barati, E., Halabian, M., Karimi, A. & Navidbakhsh, M. Numerical evaluation of stenosis location effects on hemodynamics and shear stress through curved artery. *J. Biomater. Tiss. Eng.* **4**, 358–366. 10.1166/jbt.2014.1176 (2014).

[18] Wong, K. K. L., Wu, J., Liu, G., Huang, W. & Ghista, D. N. Coronary arteries hemodynamics: Effect of arterial geometry on hemodynamic parameters causing atherosclerosis. *Med. Biol. Eng. Comput.* **58**, 1831–1843. 10.1007/s11517-020-02185-x (2020). 32519006 10.1007/s11517-020-02185-x

[19] Malvè, M. *et al.* Tortuosity of coronary bifurcation as a potential local risk factor for atherosclerosis: CFD Steady state study based on in vivo dynamic CT measurements. *Ann. Biomed. Eng.* **43**, 82–93. 10.1007/s10439-014-1056-y (2015). 24986333 10.1007/s10439-014-1056-y PMC7574594

[20] Shang, K. *et al.* Arterial tortuosity and its correlation with white matter hyperintensities in acute ischemic stroke. *Neural Plast.* **2022**, 4280410. 10.1155/2022/4280410 (2022). 35369646 10.1155/2022/4280410 PMC8970938

[21] Yang, W. J., Wong, K. S. & Chen, X. Y. Intracranial atherosclerosis: From microscopy to high-resolution magnetic resonance imaging. *J. Stroke* **19**, 249–260. 10.5853/jos.2016.01956 (2017). 28877564 10.5853/jos.2016.01956 PMC5647638

[22] Gutierrez, J. *et al.* Determinants of cerebrovascular remodeling: Do large brain arteries accommodate stenosis?. *Atherosclerosis* **235**, 371–379. 10.1016/j.atherosclerosis.2014.05.925 (2014). 24929285 10.1016/j.atherosclerosis.2014.05.925 PMC4121968

[23] Kim, B. J. *et al.* Vascular tortuosity may be associated with cervical artery dissection. *Stroke* **47**, 2548–2552. 10.1161/Strokeaha.116.013736 (2016). 27531344 10.1161/STROKEAHA.116.013736

[24] Ryu, J. *et al.* Intracranial arterial tortuosity according to the characteristics of intracranial aneurysms. *World Neurosurg.* **120**, e1185–e1192. 10.1016/j.wneu.2018.09.034 (2018). 30236811 10.1016/j.wneu.2018.09.034

[25] Zhou, L. *et al.* Plaque features and vascular geometry in basilar artery atherosclerosis. *Medicine (Baltimore)* **99**, e19742. 10.1097/MD.0000000000019742 (2020). 32358348 10.1097/MD.0000000000019742 PMC7440206

[26] Klis, K. M., Krzyzewski, R. M., Kwinta, B. M., Stachura, K. & Gasowski, J. Tortuosity of the internal carotid artery and its clinical significance in the development of aneurysms. *J. Clin. Med.*10.3390/jcm8020237 (2019). 30759737 10.3390/jcm8020237 PMC6406528

[27] Chen, Y. C. *et al.* Correlation between internal carotid artery tortuosity and imaging of cerebral small vessel disease. *Front. Neurol.*10.3389/fneur.2020.567232 (2020). 33193005 10.3389/fneur.2020.567232 PMC7642469

[28] Ha, S. H., Kim, B. J., Ryu, J. C., Bae, J. H. & Kim, J. S. Basilar artery tortuosity may be associated with early neurological deterioration in patients with pontine infarction. *Cerebrovasc. Dis.* **51**, 594–599. 10.1159/000522142 (2022). 35240597 10.1159/000522142

[29] Kashyap, V. *et al.* Accuracy of vascular tortuosity measures using computational modelling. *Sci. Rep.* **12**, 865. 10.1038/s41598-022-04796-w (2022). 35039557 10.1038/s41598-022-04796-w PMC8764056

