# Supplementary material for: Global intracranial arterial tortuosity is associated with intracranial atherosclerotic burden
Source: Sci Rep. 2024 May 17;14:11318. doi: 10.1038/s41598-024-61527-z (PMC11636879; doi:10.1038/s41598-024-61527-z)
Supplement: Supplementary file 1 — Supplementary Information. [file 41598_2024_61527_MOESM1_ESM.docx]

# Supplementary Materials

**Global intracranial arterial tortuosity is associated with intracranial atherosclerotic burden**

Mi-Yeon Eun, Ha‑Na Song, Jong‑Un Choi, Hwan‑Ho Cho, Hyung Jun Kim, Jong-Won Chung, Tae-Jin Song, Jin-Man Jung, Oh‑Young Bang, Gyeong‑Moon Kim, Hyunjin Park, David S Liebeskind, Woo-Keun Seo

Supplementary Methods

Supplementary Table 1. The mean curvature of the intracranial arteries

Supplementary Table 2. Univariable and multivariable analyses for ICAS burden prediction

Supplementary Figure 1. Graphical description of the brain vessel curvature.

Supplementary Figure 2. Subgroup analysis of the effect of GTI on intracranial atherosclerotic burden.

**Supplementary Methods**

**Quantitative Analysis of Vessel Geometric Features**

An analysis of vessel geometric features was performed using an in-house vessel analysis software, and the methods have been reported previously.^1^ Briefly, raw data were input in DICOM format, and a three-dimensional (3D) angiographic map was extracted using the region-growing method and converted into a Neuroimaging Informatics Technology Initiative format. Thus, using a vessel modeling toolkit, vessel isosurfaces were dissected. Based on the respective vertices of their isosurfaces, a continuous 3D space was divided into a uniform myriad of cells. Thereafter, the major arterial centerline was extracted from the boundary surface of each cell. Finally, cerebral arteries were reconstructed into spots (the basic unit with a 0.280-mm interval on the arterial centerline), segments, chunks, and branch units. Quantified vessel geometric features were then extracted: cross-sectional area, maximum inscribed spherical radius, minimum and maximum diameter, maximum to minimum radius ratio, surface area, distortion, curvature, and luminal (hydraulic) circularity. Each feature was presented as the mean and standard deviation values in specific arterial segments.

Three seed points on the internal carotid artery (ICA) and basilar artery (BA) were marked in the TOF image and the region-growing-based brain vessel mask segmentation was conducted. A single investigator (SHN) reviewed the binary vessel mask, verified by a certified vascular neurologist (SWK). Using a marching cube algorithm, surface nodes and edges were calculated and adjusted to isotropic triangles via spatial interpolation.^2^ 3D Voronoi diagram of surface was computed and the edges were selected as centerline candidates,^3^ and we tracked from the ICA/BA base to automatically detect vessel endpoints to determine the final centerline. The final centerline was spatially resampled into 0.2841-mm interval which is the MRA resolution.

All the above processes were performed using the open-source Python library VMTK (http://www.vmtk.org),^4^ simple insight toolkit (SITK, https://sitk.org),^5^ and sci-kit image (skimage, https://scikit-image.org).^6^

**Reference**

1. Hong, S.W. *et al.* Automated in-depth cerebral arterial labelling using cerebrovascular vasculature reframing and deep neural networks. *Sci Rep* **13,** 3255. https://doi.org/10.1038/s41598-023-30234-6 (2023).

2. Heiden, W., Goetze, T. & Brickmann, J. Fast generation of molecular surfaces from 3D data fields with an enhanced “marching cube” algorithm. *Journal of Computational Chemistry* **14,** 246-250. https://doi.org/https://doi.org/10.1002/jcc.540140212 (1993).

3. Grelard, F., Baldacci, F., Vialard, A. & Domenger, J.P. New methods for the geometrical analysis of tubular organs. *Med Image Anal* **42,** 89-101. https://doi.org/10.1016/j.media.2017.07.008 (2017).

4. Izzo, R., Steinman, D., Manini, S. & Antiga, L. The Vascular Modeling Toolkit: A Python Library for the Analysis of Tubular Structures in Medical Images. *Journal of Open Source Software* **3,** 745. https://doi.org/10.21105/joss.00745 (2018).

5. Lowekamp, B.C., Chen, D.T., Ibanez, L. & Blezek, D. The Design of SimpleITK. *Front Neuroinform* **7,** 45. https://doi.org/10.3389/fninf.2013.00045 (2013).

6. van der Walt, S. *et al.* scikit-image: image processing in Python. *PeerJ* **2,** e453. https://doi.org/10.7717/peerj.453 (2014).

Supplementary Table 1. The mean curvature of the intracranial arteries

| Mean curvature | Median (IQR) |
| --- | --- |
| R ICA | 0.25 [0.23, 0.27] |
| L ICA | 0.25 [0.23, 0.27] |
| R bMCA | 0.26 [0.24, 0.29] |
| L bMCA | 0.27 [0.24, 0.29] |
| R bACA | 0.29 [0.26, 0.32] |
| L bACA | 0.29 [0.26, 0.32] |
| R dMCA | 0.29 [0.27, 0.30] |
| L dMCA | 0.28 [0.27, 0.30] |
| R dACA | 0.29 [0.27, 0.31] |
| L dACA | 0.29 [0.27, 0.31] |
| BA | 0.20 [0.18, 0.23] |
| R VA | 0.25 [0.22, 0.33] |
| L VA | 0.24 [0.21, 0.28] |
| R bPCA | 0.29 [0.27, 0.31] |
| L bPCA | 0.28 [0.27, 0.30] |
| R dPCA | 0.33 [0.30, 0.37] |
| L dPCA | 0.33 [0.30, 0.37] |
| R CbllA | 0.32 [0.29, 0.35] |
| L CbllA | 0.32 [0.29, 0.36] |

IQR, interquartile range; R, right; ICA, internal carotid artery; L, left; bMCA, basal middle cerebral artery; dMCA, distal middle cerebral artery; bACA, basal anterior cerebral artery; dACA, distal anterior cerebral artery; BA, basilar artery; VA, vertebral artery; bPCA, basal posterior cerebral artery; dPCA, distal posterior cerebral artery; CbllA, cerebellar artery.

Supplementary Table 2. Univariable and multivariable analyses for ICAS burden prediction

|  | Common OR | P-value | Adjusted OR* | P-value |
| --- | --- | --- | --- | --- |
| Age (/10 years) | 1.26 (1.10–1.44) | 0.001 | 1.06 (0.88–1.28) | 0.528 |
| Male | 0.91 (0.64–1.28) | 0.589 | 1.12 (0.63–1.99) | 0.698 |
| BMI | 1.05 (1.00–1.10) | 0.063 | 1.02 (0.97–1.09) | 0.41 |
| Height | 0.97 (0.96–0.99) | 0.004 | 0.97 (0.94–1.00) | 0.059 |
| SBP (/10 mmHg) | 1.04 (0.98–1.10) | 0.187 |  |  |
| DBP (/10 mmHg) | 0.88 (0.79–0.97) | 0.008 | 0.94 (0.83–1.06) | 0.299 |
| Hypertension | 2.23 (1.50–3.30) | <0.001 | 1.66 (1.05–2.65) | 0.032 |
| Diabetes mellitus | 2.11 (1.49–3.00) | <0.001 | 1.71 (1.14–2.55) | 0.009 |
| Dyslipidemia | 1.38 (0.98–1.95) | 0.065 | 0.79 (0.51–1.2) | 0.27 |
| Current smoking | 0.79 (0.53–1.18) | 0.25 |  |  |
| Atrial fibrillation | 0.62 (0.39–0.98) | 0.042 | 0.92 (0.47–1.78) | 0.8 |
| Congestive heart failure | 0.81 (0.26–2.47) | 0.705 |  |  |
| Peripheral arterial disease | 7.83 (1.44–42.57) | 0.017 | 4.13 (0.67–25.45) | 0.127 |
| Previous stroke or TIA | 2.06 (1.38–3.06) | <0.001 | 1.79 (1.11–2.88) | 0.016 |
| Coronary artery disease | 2.49 (1.49–4.16) | <0.001 | 1.51 (0.82–2.78) | 0.183 |
| Aortic arch complex atheroma | 9.95 (1.92–51.5) | 0.006 | 7.01 (1.14–42.92) | 0.035 |
| Hemoglobin | 0.94 (0.86–1.02) | 0.112 |  |  |
| WBC | 1.00 (0.94–1.05) | 0.866 |  |  |
| Platelet (/10^3^) | 1.02 (0.14–7.18) | 0.985 |  |  |
| Fasting blood glucose (/10 mg/dL) | 1.02 (0.99–1.06) | 0.189 |  |  |
| Total cholesterol(/10 mg/dL) | 0.98 (0.94–1.01) | 0.207 |  |  |
| Triglyceride (/10 mg/dL) | 1.00 (0.99–1.02) | 0.654 |  |  |
| HDL-C (/10 mg/dL) | 0.88 (0.79–0.99) | 0.033 | 0.90 (0.79–1.03) | 0.119 |
| LDL-C (/10 mg/dL) | 0.99 (0.95–1.03) | 0.587 |  |  |
| hs-CRP (mg/dL) | 0.97 (0.90–1.05) | 0.504 |  |  |
| TOAST |  |  |  |  |
| SVO (ref) | 1.00 |  | 1.00 |  |
| LAA | 3.23 (2.06–5.06) | <0.001 | 2.4 (1.48–3.88) | <0.001 |
| CE | 0.77 (0.47–1.27) | 0.302 | 0.6 (0.31–1.18 | 0.14 |
| ODE | 0.60 (0.21–1.75) | 0.353 | 0.37 (0.11–1.18) | 0.092 |
| UDE | 1.45 (0.90–2.34) | 0.126 | 1.18 (0.70–1.99) | 0.543 |
| Prior antiplatelet agents use | 1.92 (1.37–2.69) | <0.001 | 0.92 (0.6–1.44) | 0.727 |
| Prior statin use | 1.74 (1.23–2.45) | 0.002 | 1.21 (0.77–1.91) | 0.409 |
| Initial NIHSS | 1.07 (1.02–1.12) | 0.003 | 1.07 (1.01–1.12) | 0.014 |
| Premorbid mRS | 1.22 (0.97–1.52) | 0.084 | 0.93 (0.72–1.21) | 0.607 |
| GTI | 1.35 (1.14–1.59) | 0.001 | 1.33 (1.09–1.62) | 0.005 |
| Area_m_ | 1.09 (0.92–1.30) | 0.306 | 1.12 (0.92–1.37) | 0.262 |

* Adjusted for age, sex, variable with p-value <0.1 in the univariate analysis, and area_m_.

ICAS, intracranial atherosclerosis; SD, standard deviation; BMI, body mass index; SBP, systolic blood pressure; DBP, diastolic blood pressure; TIA, transient ischemic attack; WBC, white blood cell; HDL-C, high-density lipoprotein cholesterol; LDL-C, low-density lipoprotein cholesterol; hs-CRP, high-sensitivity c-reactive protein; TOAST, Trial of Org 10,172 in Acute Stroke Treatment classification; LAA, large artery atherosclerosis; CE, caredioembolism; SAO, small artery occlusion; ODE, other determined etiology; UDE, undetermined etiology; mRS, modified Rankin Scale; NIHSS, National Institutes of Health Stroke Scale; Area_m,_ mean area.

Supplementary Figure 1. Graphical description of the brain vessel curvature. (a) Curvature of a virtual cerebrovascular artery. The colors along the centerline indicate different curvature values. Two circles are positioned to represent the osculating circle at a specific point on the vessel. The color of these circles and the accompanying arrow approximate the relative curvature. (b) Curvature of a spline curve (upper). The colors along the spline curve display changing curvature values. Corresponding spatial variation in the spline curvature is shown below (lower).


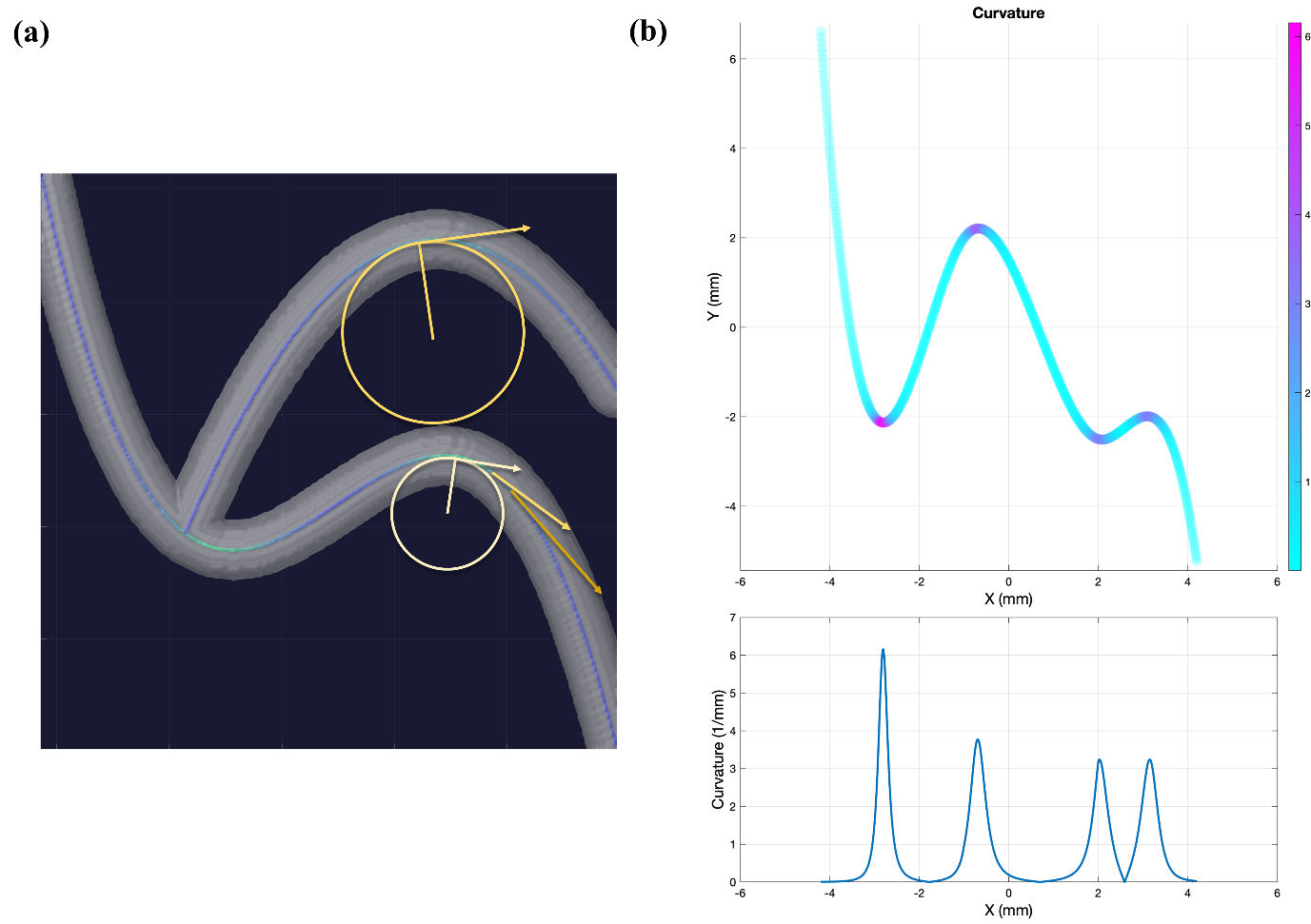


Supplementary Figure 2. Subgroup analysis of the effect of GTI on intracranial atherosclerotic burden.


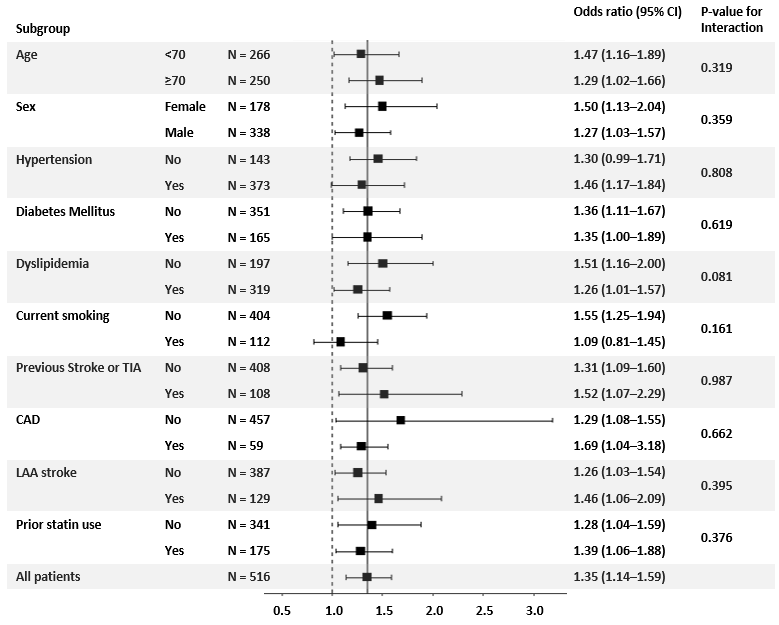


TIA, transient ischemic attack; CAD, coronary artery disease; LAA, large artery atherosclerosis; CI, confidence interval.
